# Quality of life and related factors of nursing home residents in Singapore

**DOI:** 10.1186/s12955-016-0503-x

**Published:** 2016-07-28

**Authors:** Pei Wang, Philip Yap, Gerald Koh, Jia An Chong, Lucy Jennifer Davies, Mayank Dalakoti, Ngan Phoon Fong, Wei Wei Tiong, Nan Luo

**Affiliations:** 1 Saw Swee Hock School of Public Health, National University of Singapore, 12 Science Drive 2, Tahir Foundation Building, Singapore, Singapore; 2 Khoo Teck Puat Hospital, Singapore, Singapore; 3 Ministry of Health, Singapore, Singapore; 4 Singapore General Hospital, Singapore, Singapore; 5 National University Health System, Singapore, Singapore

**Keywords:** Quality of life, Nursing home, Risk factors, Singapore

## Abstract

**Background:**

Litter is known about the well-being of nursing home (NH) residents in Singapore. This study aimed to identify predictors of self-reported quality of life (QOL) of NH residents in Singapore.

**Methods:**

In face-to-face interviews, trained medical students assessed each consenting resident recruited from 6 local NHs using a modified Minnesota QOL questionnaire, and rating scales and questions assessing independence, cognitive function, depression, and communication. Predictors of residents’ QOL in five aspects (comfort, dignity, food enjoyment, autonomy, and security) were identified using the censored least absolute deviations (CLAD) models.

**Results:**

A total of 375 residents completed the interviews. A higher score on comfort was negatively associated with major depression while a higher score on dignity was positively associated with no difficulty in communication with staff. Higher scores in food enjoyment were negatively associated with major depression and poorer cognitive function. Higher scores in autonomy were negatively associated with major depression, greater dependence, and difficulty in communication with staff. A higher score on security were negatively associated with major depression.

**Conclusion:**

It appears that depression and difficulty in communication with staff are the two main modifiable risk factors of poor quality of life of local NH residents.

## Background

Singapore has a rapidly aging population. It is estimated that 20 % of its population will be above 65 years by 2030 [1]. The trend will be accompanied an increasing demand for nursing homes (NHs) providing intermediate and long-term care to chronically-sick elderly who do not have families or caregivers to look after them at home, or are unable to provide the level of nursing care required [2]. The Ministry of Health projects to increase the number of beds available in NHs by 70 % to 15,600 by 2020 to meet the demand [3], and NHs will play a central role in the future provision of custodial, social and healthcare for seniors in Singapore [4].

NHs in Singapore are run by Voluntary Welfare Organizations (VWOs) or private operators. As of 2013, there were 66 NHs in Singapore providing 10,652 beds [5]. NH residents in Singapore are typically frail with multiple health issues: 52 % have mental problems, 53 % visual impairment, 46 % hypertension, 42 % stroke, 41 % severely dependent in the Activities of Daily Living (ADL), and 48 % cognitively impaired [6]. Based on the findings of a study just over a decade ago, the clinical care to NH residents was not optimal as suggested by a high prevalence of malnutrition (22 %) [7], functional decline (35 %) [7], mood and sleep problems (50 % to 70 %) [7]. These objective indicators may suggest a compromised quality-of-life (QOL) in the NH residents since quality of care can affect perceived QOL of the residents [8].

QOL is multi-dimensional with objective and subjective constructs, although the latter is often given greater priority. As such, QOL often refers to individuals’ life satisfaction, happiness, and morale. For example, a World Health Organization workgroup defined QOL as “individuals’ perceptions of their positions in life in the context of the culture and value systems in which they live and in relation to their goals, expectations, standards and concerns” [9]. Optimizing the QOL of NH residents is germane to healthcare professionals and caregivers in NHs. However, improving QOL requires a comprehensive and in-depth understanding of the primary dimensions of QOL and related factors among NH residents. Previous studies on QOL of NH residents have shown that dignity, spiritual well-being, food enjoyment, leisure activities, and independence are amongst the most important aspects of QOL [10, 11]. Predictors of poor QOL in NH residents have also been identified. These include modifiable factors such as ADL-dependency [12], physical impairment [13], major depressive disorder [12], neuropsychiatric symptoms (i.e. behavior problems) of dementia [14], and poor socioeconomic status and social support [15, 16]; and non-modifiable factors such as cognitive impairment [12], female [17], medical history of multiple comorbidities [15], for-profit NHs [18], and longer duration of stay in NH [16].

In Singapore, there has been little work undertaken to holistically assess the QOL of residents in NHs or identify its predictors, although some studies have examined isolated QOL domains such as the prevalence of pain (42.9 %-48.7 %) [19]. To aid policy makers develop appropriate strategies to improve QOL of NH residents in Singapore, this study aimed to systematically evaluate the self-reported QOL of the residents and identify its predictors.

## Methods

### Study sample

The study was approved by the Institutional Review Board of National University of Singapore (NUS) and conducted during 31 January 2012 to 8 February 2012. Participants were recruited from six local NHs run by VWOs; attempts to recruit residents from private NHs were not successful because no private NHs agreed to conduct the study in their premises. The inclusion criteria for the subjects were: 1) age ≥55 years; 2) residence in the NH for at least 3 months; 3) able to communicate views and opinions coherently; and 4) able to give informed consent to participate. NH residents who were uncommunicative or unable to respond meaningfully due to physical or cognitive impairment were excluded. A list of potential eligible NH residents was provided by a nurse manager from each NH to form the sampling frame. The residents’ ability to communicate was assessed using three screening questions: “What is your name?”; “What is your age?”; and “Where are you now?” If a resident answered irrelevantly to any of the questions (e.g. responded ‘very good’ to ‘What is your age?’) despite asking thrice, the resident would be assessed to be unable to communicate meaningfully and excluded. All eligible residents were informed both verbally and in writing, and informed consent was obtained from all participants.

### Data collection

Consenting NH residents were interviewed face-to-face by 16 pairs of NUS medical students. They had been trained to strictly adhere to the phrasing of the questions, to avoid excessive prompting of the residents, and to pay attention to non-verbal cues that may signify distress or withdrawal of consent, especially in residents with dementia who may not be able to express themselves adequately.

A questionnaire with various rating scales and questions was administered to the NH residents, with each interview session lasting between 15–30 min. The interviews were conducted in English, Mandarin, Malay, or one of the two main local dialects Hokkien and Cantonese. Multi-lingual translators were employed if the primary interviewer was not conversant with the participant’s language or dialect. The NH residents’ demographic (e.g. age, education) and clinical information (e.g. history of medical conditions, current medications) was retrieved from their medical records.

### Measures

The rating scales used included the modified Minnesota questionnaire for assessing QOL, the Abbreviated Mental Test (AMT) for cognitive function, the Structural Clinical Interview (SCID) for depression, and the Katz Index of Independence in ADL.

The QOL scale used was designed based on the University of Minnesota study [20] which published a 66-item questionnaire examining 11 domains of QOL including comfort, security, functional competence, relationship, enjoyment, meaningful activities, dignity, privacy, autonomy, and spiritual well-being. The questionnaire has been well-validated for NH residents and has an abbreviated and validated version containing 34-item in 9 domains [20]. The abbreviated version was chosen to be adapted and tested in a pilot study, which was conducted on a total of 19 residents selected by the nursing directors of four NHs. The pilot study aimed to identify potential difficulties in questionnaire administration and distinguish the top 5 domains that were most relevant to local NH residents. This would help to shorten the length of the questionnaire and minimize burden to the residents while maintaining the adequacy of questionnaire for application to the local residents. The domains identified were security (79.6 %), dignity (77.8 %), food enjoyment (71.9 %), autonomy (70.2 %), and comfort (68.4 %). All the 18 items in the 5 domains (each domain has 3 or 4 items) were extracted from the questionnaire to generate a new questionnaire. Moreover, the items were revised based on the pilot study to minimize ambiguity and improve relevance to the local context. For example, ‘How often are you too cold here?’ was modified to ‘During your stay here, do you feel too hot or too cold?’ to better reflect the tropical climate in Singapore. The response scale was also modified from a 4-point Likert format to a 2-point format (1=‘mostly yes’, 2=‘mostly no’), both of which were recommended [20]. Meanwhile, a ‘not applicable’ option was also added to the response scale as some questions were found to be irrelevant for several NH residents in the pilot study.

The AMT is a 10-item scale for measuring the residents’ cognitive function [21]. It has a total score of 10 and the cut-off was set at the clinically validated score of 6/10. NH residents who have a score of ≤6 were assessed as having cognitive impairment.

The SCID which uses the Diagnostic and Statistical Manual of Mental Disorders, fourth edition (DSM-IV) criteria was chosen as the tool to assess depression in the study [22]. It is the gold standard diagnostic tool for measuring depression in the clinical setting and consists of 9 questions evaluating the presence of symptoms associated with depression. The cut-off value for major depression was set at ≥5/9. Minor depression was defined as the presence of 2 to 4 depressive symptoms.

The Katz Index of Independence in ADL was used to assess functional limitations of ADL in 6 aspects including bathing, dressing, toileting, transferring, continence, and feeding [23]. It adopted a dichotomous format, with 1 point for each ADL if the NH resident was independent (defined as requiring no assistance from the NH staff), and 0 point if the NH resident required any form of assistance. The cut-off value was determined at 3/6: those who were dependent in 0–3 ADLs and those who were independent in 4–6 ADLs. The cut-off value is also consistent with the definition of disability used in Eldershield which is a local insurance scheme providing basic financial protection for persons who need long term care in Singapore.

The QOL scale, AMT, SCID, and Katz were translated into Mandarin using forward-translation and back-translation which were done independently by different persons to ensure accuracy and appropriateness of the wording.

The questionnaire also included questions to determine whether the NH residents suffered from a lack of social contact, had a history of falls or being restrained, and had experienced difficulty in communication with staff in the NH.

### Data analysis

Cronbach’s alpha, a measure of internal consistency, was calculated to assess the reliability of the five QOL scales as well as the AMT and Katz index of independence in ADL. A Cronbach’s alpha value ≥ 0.7 indicates a satisfactory level of reliability.

To identify predictors of NH residents’ QOL, 16 variables were examined. All of them were coded as categorical variables including age (<80 years vs. > = 80 years); gender; race (Chinese vs. non-Chinese); marital status (no partner vs. with partner); education (no or low education vs. higher education [secondary school and above]); religion (religion vs. no religion); length of stay (<=2 years vs. >2 years); depression (no depression, minor depression, and major depression), number of comorbidities (> = 3 vs. <3); cognitive functioning (AMT < =6 vs. AMT >6); functional impairment (ADL score < =3 vs. ADL score >3); social contact (no or lack of social contact vs. with social contact [one visitor visit at least once a month]); history of being restrained since admitted into NH (with vs. without); history of falls since admitted into NH (with vs. without); communication with staff (no difficulty vs. difficulty); and study sites (the six NHs). The majority was identified as predictors of QOL of NH residents previously. Sociodemographic factors such as education, marital status, and religion were also examined in prior studies [15, 16]; race was added in our study since Singapore is a multiethnic country. As NH staff tend to be more cautious with residents with a history of being restrained or falls and thus afford them less autonomy, the two factors were also examined. Study sites were explored as evidence has suggested that residents’ satisfaction with the NH was a significant predictor of high QOL [10].

The relationship between NH residents’ QOL and the variables was explored in each of the five domains of the QOL scale. Five multivariate regression models were used to identify factors significantly associated with the five domain scores, respectively. The domain scores were calculated by aggregating the item scores within the domain, ranging from 3 to 6 or 4 to 8. Reverse scoring was applied whenever appropriate to make higher scores consistently indicating better QOL. As the QOL data exhibited ceiling effects and heteroscedasticity, we adopted the censored least absolute deviations (CLAD) estimator [24] to estimate the coefficients of the regression model. The CLAD model does not require distributional assumptions or homoscedasticity assumption of residuals, and is robust to censoring [24]. Empirical evidence has suggested that it outperforms the OLS model and the Tobit model in the face of non-normality, heteroscedasticity, and censoring [25–27].

## Results

A total of 597 NH residents comprised the sampling frame of which 222 (36.9 %) were excluded because they (1) were uncommunicative (n = 83); (2) declined participation (n = 73); (3) unable to respond meaningfully (n = 41); (4) aged <55 years (n = 14); or (5) had stayed in the NH for <3 months (n = 11). As a result, 375 residents comprised the final sample.

The mean age of the residents was 77.3 (standard deviation [SD] =10.3) years, with female comprising 53.9 %. The majority was ethnic Chinese (86.9 %) and many residents spoke local dialects (i.e. Hokkien and Cantonese) (53.4 %). Half of the residents (50.4 %) were functionally dependent, and 62.9 % residents had stayed in the NH for more than two years. A significant proportion (40.3 %) had poor cognitive function (AMT ≤ 6) while the majority (78.9 %) was not depressed. Most of the residents (82.4 %) reported a visitor at least once a month and were able to communicate with staff (86.7 %) in the NH (Table 1).

**Table 1:** Characteristics of nursing home residents (n = 375)

	N (%)
Age	
< 80	206 (54.9)
≥ 80	169 (45.1)
Gender	
Male	173 (46.1)
Female	202 (53.9)
Race	
Chinese	326 (86.9)
Non-Chinese	49 (13.1)
Marital status	
With partner	69 (18.4)
No partner	306 (81.6)
Education	
No or low education	273 (72.8)
Higher education	102 (27.2)
Religion	
Religion	339 (90.4)
No religion	36 (9.6)
Language spoke	
English	78(20.8)
Mandarin	72(19.2)
Hokkien	164(43.7)
Cantonese	36 (9.6)
Others	25 (6.7)
Length of stay	
< 2 years	139 (37.1)
≥ 2 years	236 (62.9)
Medication	
Non-parenteral/non-oral medication	8 (2.1)
Parenteral/oral medication	367 (97.9)
Depression	
No depression	296 (78.9)
Minor depression	54 (14.4)
Major depression	25 (6.7)
Comorbidities	
< 3	94 (25.1)
≥ 3	281 (74.9)
ADL status	
Independent in 0–3 ADLs	189 (50.4)
Independent in 4–6 ADLs	186 (49.6)
AMT score	
AMT ≤6	115 (40.3)
AMT >6	255 (59.7)
Social contact	
With social contact	309 (82.4)
No or lack of social contact	66 (17.6)
History of being restrained	
Yes	45 (12.1)
No	327 (87.9)
History of falls	
Yes	85 (22.9)
No	287 (77.2)
Difficulty in communication with staff	
No difficulty	325 (86.7)
Difficulty	50 (13.3)
Nursing homes	
1	66 (17.6)
2	55 (14.7)
3	100 (26.7)
4	54 (14.4)
5	53 (14.1)
6	47 (12.5)

The residents’ responses to the 18 QOL questions in 5 domains are shown in Table 2. All questions had most residents reporting positive answers, ranging from 52.5 % (QOL15) to 88.5 % (QOL18) (Table 2). The mean domain scores were 7.0 (max = 8, SD = 1.1) for comfort, 6.7 (max = 8, SD = 0.9) for dignity, 5.2 (max = 6, SD = 0.9) for food enjoyment, 6.6 (max = 8, SD = 1.1) for autonomy, and 4.8 (max = 6, SD = 0.6) for security, respectively.

**Table 2:** Quality of life of nursing home residents

No.	Question	Mostly Yes (%)	Mostly No (%)	Not Applicable (%)	Domain Score (SD)
Comfort					7.0 (1.1)
QOL1	During your stay here, do you feel too hot or too cold?	28.5	70.4	1.1	
QOL2	During your stay here, do you feel pain because you are in the same position for too long?	21.1	77.6	1.3	
QOL3	During your stay here, do you feel pain anywhere?	29.3	70.4	0.3	
QOL4	Are you bothered by noise in your room?	24.3	75.5	0.3	
Dignity					6.7 (0.9)
QOL5	Do staff here treat you politely?	81.6	16.5	1.9	
QOL6	Do you feel that you are treated with respect here?	76.5	21.1	2.4	
QOL7	Do staff here handle you roughly while caring for you?	20.8	77.3	1.9	
QOL8	Do staff here respect your modesty? e.g. closing the door when bathing you or closing the curtains when you are changing	76.8	15.2	8.0	
Food enjoyment					5.2(0.9)
QOL9	Do you like the food here?	68.8	24.5	6.7	
QOL10	Do you enjoy eating with the other residents?	70.7	19.2	10.1	
QOL11	What is your favorite food? Can you get it here?	54.9	31.2	13.9	
Autonomy					6.6 (1.1)
QOL12	Can you go to bed at the time you want?	79.2	19.7	1.1	
QOL13	Can you get up in the morning at the time you want?	61.6	36.8	1.6	
QOL14	Can you decide what clothes to wear?	53.6	41.3	5.1	
QOL15	If you tell the staff about something you do not like, do they change it?	52.5	23.5	24.0	
Security					4.8 (0.6)
QOL16	Do you feel that your belongings/things are safe at this nursing home?	63.5	32.8	3.7	
QOL17	Do your clothes get lost or damaged in the laundry?	17.9	69.3	12.8	
QOL18	Do you feel safe and secure here?	88.5	8.1	2.4	

*QOL* quality of life, *SD* standard deviation

Cronbach’s alpha values were consistently below 0.7 for all QOL domains: comfort (0.543), dignity (0.654), food enjoyment (0.458), autonomy (0.443), and safety (0.545). On the other hand, the alpha value was 0.791 for the Katz index of independence in ADL and 0.829 for AMT.

Table 3 presents the results of 5 CLAD regression analyses for the potential predictors of the 5 QOL domain scores. A higher score on comfort was negatively associated with major depression while a higher score on dignity was positively associated with no difficulty in communication with staff. Higher scores in food enjoyment were negatively associated with major depression and poor cognitive function. Higher scores in autonomy were negatively associated with depression and ADL dependence but positively associated with no difficulty in communication with staff. Higher scores in security were negatively associated with major depression. No other demographic or clinical characteristic significantly predicted these QOL domain scores.

**Table 3:** Multivariate regression analysis of predicators of quality of life of nursing home residents using CLAD model

	Comfort		Dignity		Food Enjoyment		Autonomy		Security	
	Coefficients	CI	Coefficients	CI	Coefficients	CI	Coefficients	CI	Coefficients	CI
Age										
< 80	0.05	(−0.44, 0.54)	0.14	(−0.10, 0.38)	0.13	(−0.55, 0.80)	0.08	(−0.31, 0.46)	0.03	(−0.17, 0.23)
≥ 80										
Gender										
Male	0.17	(−0.34, 0.69)	0.10	(−0.14, 0.34)	−0.42	(−1.10, 0.26)	0.26	(−0.74, 0.21)	0.11	(−0.09, 0.31)
Female										
Race										
Chinese	0.47	(−0.13, 1.08)	−0.06	(−0.40, 0.27)	0.04	(−0.76, 0.84)	0.58	(−0.01, 1.18)	0.13	(−0.16, 0.41)
Non-Chinese										
Marital Status										
With partner	−0.46	(−1.11, 0.18)	0.04	(−0.25, 0.34)	−0.25	(−1.19, 0.69)	0.13	(−0.51, 0.78)	−0.04	(−0.30, 0.21)
No partner										
Education										
No or low education	0.54	(−0.01, 1.08)	0.09	(−0.17, 0.35)	0.17	(−0.36, 1.37)	0.13	(−0.33, 0.60)	−0.12	(−0.34, 0.11)
Higher education										
Religion										
Religion	0.24	(−0.39, 0.88)	−0.06	(−0.43, 0.32)	−0.13	(−1.26, 0.90)	0.11	(−0.69, 0.91)	−0.02	(−0.37, 0.32)
No religion										
Length of stay										
< 2 years	0.21	(−0.43, 0.85)	−0.11	(−0.35, 0.13)	0.06	(−0.88, 1.26)	−0.26	(−0.74, 0.21)	0.06	(−0.14, 0.27)
≥ 2 years										
Depression										
No depression	**1.67**	(0.71, 2.62)	**0.75**	(0.26, 1.23)	**1.21**	(0.13, 2.51)	**0.68**	(0.02, 1.38)	**0.48**	(0.07, 0.89)
Minor depression	**1.31**	(0.29, 2.33)	0.40	(−0.15, 0.95)	**1.13**	(0.30, 2.54)	0.43	(−0.56, 1.43)	0.31	(−0.15, 0.77)
Major depression										
Comorbidities										
< 3	−0.16	(−0.66, 0.33)	−0.09	(−0.35, 0.17)	0.13	(−0.27, 1.37)	0.04	(−0.41, 0.48)	−0.17	(−0.39, 0.04)
≥ 3										
ADL status										
Independent in 0–3 ADLs	−0.32	(−0.80, 0.16)	−0.16	(−0.40, 0.07)	−0.08	(−0.80, 0.34)	**−0.32**	(−0.80, −0.16)	−0.19	(−0.38, 0.01)
Independent in 4–6 ADLs										
AMT score										
AMT ≤6	−0.10	(−0.54, 0.34)	0.07	(−0.18, 0.31)	**−0.42**	(−1.06, −0.13)	−0.06	(−0.50, 0.38)	0.14	(−0.07, 0.34)
AMT >6										
Social contact										
With social contact	−0.33	(−0.96, 0.31)	0.01	(−0.29, 0.31)	0.29	(−0.46, 1.04)	0.08	(−0.51, 0.66)	0.02	(−0.24, 0.27)
No or lack of social contact										
History of being restrained										
Yes	−0.42	(−1.12, 0.27)	−0.03	(−0.40, 0.33)	−0.25	(1.15, −0.44)	−0.17	(−0.96, 0.62)	−0.14	(−0.47, 0.18)
No										
History of falls										
Yes	−0.27	(−0.87, 0.27)	−0.19	(−0.48, 0.09)	0.08	(−0.34, 1.01)	0.25	(−0.23, 0.72)	0.04	(−0.20, 0.28)
No										
Difficulty in communication with staff										
No difficulty	−0.12	(−0.80, 0.57)	**0.95**	(0.60, 1.29)	0.21	(−0.84, 1.31)	**0.81**	(0.24, 1.38)	0.50	(0.20, 0.80)
Difficulty										
Nursing homes										
1	−0.50	(−1.36, 0.36)	**−0.69**	(−1.10, −0.27)	−0.71	(−2.02, 0.60)	0.43	(−0.38, 1.25)	0.10	(−0.23, 0.44)
2	0.08	(−0.84, 0.99)	−0.09	(−0.52, 0.34)	−0.13	(−1.43, 1.18)	0.57	(−0.21, 1.35)	−0.09	(−0.43, 0.26)
3	0.50	(−0.78, 0.88)	**−0.36**	(−0.74, −0.02)	−0.33	(−1.43, 0.76)	−0.31	(−0.99, 0.38)	0.13	(−0.21, 0.46)
4	−0.46	(−1.30, 0.37)	−0.19	(−0.62, 0.24)	−0.38	(−1.65, 0.90)	**0.69**	(0.15, 1.28)	−0.02	(−0.35, 0.32)
5	−0.41	(−1.27, 0.44)	−0.04	(−0.46, 0.38)	0.08	(−1.20, 1.37)	0.30	(−0.41, 1.02)	0.21	(−0.13, 0.55)
6										
Pseudo R^2^	0.081		0.107		0.082		0.131		0.124	

*CLAD* censored least absolute deviations, *CI* confidence interval. Boldness indicates statistically significant difference at *p*<0.05

## Discussion

This study investigated the self-reported QOL of Singaporean NH residents in the 5 domains of comfort, dignity, food enjoyment, autonomy, and security. The majority of residents rated their QOL favorably as suggested by a significant proportion of positive responses in the QOL items, which may imply at least a reasonable quality of care in local NHs.

On the other hand, there were still many residents who gave less satisfactory responses, suggesting that their quality of life may have been suboptimal. In our study, more than 30 % residents stated that they could not have their favorite food (31.2 %), could not wake up at the time they desired (36.8 %) or decide what clothes they wished to wear (41.3 %). These indicate that the manpower and resources spent in the NHs may have not been adequate. It may not be possible to increase satisfaction in those domains without spending more resources. However, improvements could be made easily in some other areas. For example, about 15 % residents indicated that staff in the NHs do not respect their modesty during care (e.g. closing the door when bathing them or closing the curtains when they are changing), which can be avoided.

Residents’ QOL can also be enhanced through targeting its modifiable factors. We found potentially modifiable factors in depression, communication with staff, ADL dependence and a non-modifiable factor cognitive function to be significantly related to QOL. Specifically, residents with major depression, inability to communicate with the staff, ADL dependence, and poorer cognitive function had poorer QOL in one or more aspects.

Residents with major depression reported poorer QOL in comfort, food enjoyment, and security, which is in line with findings in extant literature [28, 29]. This is not surprising as depression impacts one’s morale which in turn affects one’s perception of comfort, security and ability to enjoy food. Conversely, a lack of comfort or security and food aversion can likewise worsen depression, resulting in a vicious circle that can have severe detrimental effects on the resident’s well-being.

A resident’s inability to communicate with NH staff was associated with lower QOL in dignity and autonomy. Language barriers between staff and residents have been exacerbated by the increasing number of foreign staff in recent years [30]. The majority of the residents could only speak local dialects such as Hokkien and Cantonese (53.4 %) while most foreign staff are unable to converse fluently in dialects. As a result of communication barriers, residents may not be able to indicate their needs, and opinions to staff. This finding is distinctive to Singapore which has an eclectic mix of ethnicity, language and culture. It is certainly imperative to draw more dialect conversant locals to the NH workforce and this can be done by increasing the benefits of staff working in NHs. Foreign staff could be put through courses in local language and dialects to improve communication between staff and residents, and potentially enhance the well-being of the residents.

Residents with lower independence in ADL had poorer QOL in autonomy. This is not surprising and in accordance with existing studies [13, 16], where NH residents who were more dependent were found to require more assistance from staff, causing loss of autonomy. In pricing safety above autonomy, QOL can often be compromised. If QOL of the residents is a priority, a balance between safety and autonomy would be needed.

Those residents who had cognitive dysfunction (i.e. AMT < =6) had lower domain score of food enjoyment. The association between poorer cognitive function and poorer QOL was supported in the literature [12]. The cognitively impaired residents are typical of dementia, who were unlikely to enjoy the food.

Overall, the study has found QOL in Singaporean NH residents to be satisfactory, and depression and difficulty in communication with staff are the two main modifiable factors that should be addressed. In addition, providing more personalized care to meet the unique needs of each resident and to afford greater resident autonomy will do well to secure better QOL for the residents. Evidently, more manpower in the NH workforce is needed, especially to draw locals to work in the NH sector [4]. This would warrant greater investment to secure better work benefits and career prospects to make the field more attractive.

Some limitations in the study are noteworthy. First, we used a culturally adapted QOL instrument which had not been formally validated in the study population. The instrument measured only five QOL concepts and had suboptimal reliability. However, our pilot study did not support the use of the original instrument which is based on a longer questionnaire using 4-point Likert response scales. This was mainly because many members of our study population were not well educated or cognitively unwell. Nevertheless, our pilot study ensured that validity of the instrument in that only QOL domains most important to our study population were kept in the questionnaire. Second, the results were based on residents in NHs run by VWOs only, which may not be generalizable to residents in private NHs. Third, as residents who were uncommunicative or unable to communicate meaningfully were excluded, the overall QOL could have been an overestimate as these residents are intuitively surmised to have a lower QOL. Fourth, interviewer bias could be an issue. Although training for the interviewers was done to ensure standardization, subtle clues to the answers or tone of voice from different interviewers may influence the residents’ answers. Finally, variations of QOL in dignity and autonomy were found across the NHs (Table 3). This observation indirectly corroborates with findings from previous studies in which QOL domains such as dignity, food enjoyment and global NH satisfaction were positively related to each other [7]. The variations could be due to differing capabilities of the NHs in manpower, infrastructure, and funding. These factors were, however, not investigated in the study due to limited resources. Future studies are necessary to explore and understand the reasons for the variations.

## Conclusions

The findings of the study have important implications for improving the quality of care in NHs of Singapore and to guide programs and interventions for the residents. Joint efforts will be necessary between the local health authorities and service providers to set and deliver higher standards of care. The recent introduction of the enhanced NH standards is a case in point and certainly a step in the right direction [31]. We hope that more of such efforts can be initiated in the near future to bring about better QOL in our NH residents.

## Abbreviations

ADL, activities of daily living; AMT, abbreviated mental test; NH, nursing home; NUS, National University of Singapore; QOL, quality of life; SCID, structural clinical interview; SD, standard deviation; VWO, Voluntary Welfare Organizations
